# Relationships of CD163 and CD169 positive cell numbers in the endometrium and fetal placenta with type 2 PRRSV RNA concentration in fetal thymus

**DOI:** 10.1186/s13567-016-0364-7

**Published:** 2016-08-05

**Authors:** Predrag Novakovic, John C. S. Harding, Andrea Ladinig, Ahmad N. Al-Dissi, Daniel J. MacPhee, Susan E. Detmer

**Affiliations:** 1 Department of Veterinary Pathology, Western College of Veterinary Medicine, University of Saskatchewan, Saskatoon, SK Canada; 2 Department of Large Animal Clinical Sciences, Western College of Veterinary Medicine, University of Saskatchewan, Saskatoon, SK Canada; 3 Department of Veterinary Biomedical Sciences, Western College of Veterinary Medicine, University of Saskatchewan, Saskatoon, SK Canada; 4 University Clinic for Swine, Department for Farm Animals and Veterinary Public Health, University of Veterinary Medicine Vienna, Vienna, Austria

## Abstract

Several routes of porcine reproductive and respiratory virus PRRSV transmission across the porcine diffuse epitheliochorial placentation have been proposed, but none have been proven. The objectives of this study were to investigate associations between numbers of CD163 and CD169 positive macrophages, cathepsin positive areolae, and type 2 PRRSV load at the maternal–fetal interface in order to examine important factors related to transplacental infection. On gestation day 85 ± 1, naïve pregnant gilts were inoculated with PRRSV (*n* = 114) or were sham inoculated (*n* = 19). At 21 days post-inoculation (dpi), dams and their litters were humanely euthanized and necropsied. Samples of the maternal–fetal interface (uterus with fully attached placenta) and fetal thymus were collected for analysis by RT-qPCR to quantify PRRSV RNA concentration. The corresponding paraffin-embedded uterine tissue sections were subjected to immunohistochemistry for PRRSV nucleocapsid N protein, CD163, CD169, and cathepsin. Our findings confirm significant increases in the numbers of PRRSV, CD163 and CD169 positive cells at the maternal–fetal interface during type 2 PRRSV infection in pregnant gilts. PRRSV load in fetal thymus was positively related to CD163^+^ cell count in endometrium and negatively related to CD163^+^ cell count in placenta, but unrelated to CD169 counts or cathepsin positive areolae. The endometrium:placenta ratio of CD163 cells, and to a lesser extent CD169 cells, was significantly associated with an increase fetal viral load in thymus. These findings suggest a more important role for CD163^+^ cells following trans-placental PRRSV infection, but dichotomous responses in endometrium and placenta for both CD163 and CD169 cells.

## Introduction

Porcine reproductive and respiratory syndrome (PRRS) continues to be the most costly disease of the North American swine industry resulting in economic losses estimated to be $664 million annually [1]. PRRS is caused by an enveloped positive-stranded RNA virus, in the genus of Arterivirus and family *Arteriviridae* [2–4]. Viral replication of porcine reproductive and respiratory syndrome virus (PRRSV) initially occurs in local permissive macrophages and then rapidly spreads to well-differentiated monocyte-derived cells, such as pulmonary alveolar macrophages (PAM), intravascular macrophages (PIM) in the lung [5–7], and macrophages in lymphoid tissue [2].

Two markers identified on the surface of permissive macrophages are CD163 entry mediator and sialoadhesin (CD169) receptor [8]. CD163 is a glycosylated membrane protein expressed almost exclusively on macrophages and monocytes [9]. As a macrophage scavenger receptor, CD163 is involved in taking up haptoglobin-hemoglobin complexes, erythroblast adhesion, innate immunity of bacteria, and binding of TNF-like inducers of apoptosis [9]. CD163 is an essential receptor for the entry and uncoating of PRRSV from the early endosomes of permissive cells [10].

Attachment of PRRSV to target cells is believed to occur through the interaction of the viral ligand GP5 and M heterodimer complex with CD169 receptor [10, 11]. CD169 receptors belong to the family of sialic acid-binding immunoglobulin-like lectins that are expressed on specific subsets of tissue macrophages found in the spleen, lymph nodes, bone marrow, liver, colon, and lungs [12]. Previously, CD169 was reported to be involved in the attachment and internalization of viruses [5] and bacteria [13], but recent studies with porcine CD169 suggest its role as an endocytic receptor in targeted delivery of toxins and antigens to macrophages [14].

Both CD169 and CD163 have been shown to be required for type 1 PRRSV infection in vitro [8]. It has also been demonstrated that both CD169 and CD163 positive macrophages are increased within the implantation sites and organs of porcine embryos and fetuses during gestation [15]. However, CD169 negative transgenic pigs infected with type 2 PRRSV have demonstrated no difference in virus replication compared to infected wildtype pigs [16], while CD163 negative transgenic pigs did not develop type 2 PRRSV infection [17]. Recent studies with type 1 PRRSV infection of pregnant sows have confirmed that all PRRSV-infected cells at the maternal–fetal interface (MFI) were also CD163 and CD169 positive [18]. Additionally, significant association between type 1 PRRSV infection and the numbers of CD169 positive cells in MFI was found [19].

While CD163 and CD169 macrophages have been explored, other potential mechanisms of transplacental infection of PRRSV have been largely uninvestigated. In pigs, the transplacental exchange of gases, micronutrients and macromolecules is accomplished by maternal hematotrophic and histotrophic nourishment of the fetus. Histotroph is a source of nutrition for the fetus provided by secretions of uterine epithelia in the dome-shaped structures over the openings of uterine glands called areolae [20]. In the areolae, secretions from superficial and deep uterine glandular epithelium, and selective transudation from maternal serum are absorbed and transported across the chorioallantois by fluid phase pinocytosis into the fetal circulation [21]. During gestation, lysosomal cysteine protease cathepsin-L, is highly expressed in the chorionic epithelium of the areolae [22]. Therefore, a potential site of transplacental infection may be areolae.

Three routes of transplacental spread of PRRSV have been discussed in the literature [23], such as direct spread from infected macrophages to epithelial cells of uterus and fetal placenta, spread of free PRRS viral particles, and migration of infected macrophages from mother to the fetus. Results of our histopathological evaluation of type 2 PRRSV infection in pregnant gilts in the third trimester of pregnancy confirmed marked inflammatory changes affecting the maternal–fetal chorionic interdigitation areas [24] and suggested potential role for inflammatory cells and resident macrophages in PRRSV infection of the fetal placenta and fetus [23]. In order to further test this hypothesis, we developed two objectives for the present study. The first objective was to evaluate the numbers of PRRSV, CD163 and CD169 positive cells in the endometrium and fetal placenta, and to test if the numbers of cathepsin positive areolae at the MFI differed between groups selected on the basis of PRRSV viral load at the MFI (negative, low, high). The second objective was to assess the relationship between PRRSV viral load in the fetal thymus compared to the numbers of CD163 and CD169 positive cells in the endometrium and fetal placenta, and cathepsin positive areolae at the MFI.

## Materials and methods

### Experimental design and selection of samples

The animal use protocol was reviewed and approved by the Animal Research Ethics Board (AREB) at the University of Saskatchewan and followed the principles established by the Canadian Council on Animal Care (permit #20110102). The experimental protocol for this study has been described in detail [25]. Briefly, on 85 ± 1 gestation day, 114 PRRSV-naïve pregnant gilts were intramuscularly and intranasally inoculated with type 2 PRRSV (1 × 10^5^ TCID_50_ total dose, NVSL 97–7895, Gen Bank Accession No. AF325691) and 19 negative control pregnant gilts were sham inoculated with minimum essential medium (Life Technologies, Burlington, Canada). At 21 days post-inoculation (dpi), dams and their litters were humanely euthanized for necropsy examination. Samples of fetal thymus and MFI (endometrium with adherent placental layers) adjacent to the umbilical stump of each fetus were collected for histology and an in-house quantitative RT-PCR analysis of PRRSV RNA concentration, as previously described [25]. From a total of 679 available MFI samples collected from live fetuses with intact uterine-placental tissue, 120 paraffin-embedded MFI samples were selected, based on the PRRSV RNA concentration in the MFI, for PRRSV, CD163, CD169, and cathepsin immunohistochemistry (IHC). Three viral load groups were formed: negative, low and high. Negative samples were RT-qPCR negative samples from non-infected gilts. Low viral load samples from infected gilts had PRRSV RNA concentration less than 2.3 log_10_ copies per mg MFI (mean 0.7 ± 0.8 sd; *n* = 40). High viral load samples from infected gilts had PRRSV RNA concentration greater than 2.8 log_10_ copies per mg MFI (mean 5.2 ± 1.2 sd; *n* = 40). The low and high samples were matched pairs from 40 infected gilts. The negative samples came from five gilts, eight samples per gilt.

### PRRSV immunohistochemistry

Five-micrometer tissue sections were prepared for IHC using proteinase K (Dako, Carpinteria, USA) antigen retrieval and Background Punisher blocking reagent (Biocare Medical, Concord, USA). The wash buffer was 0.05 M Tris-buffered saline (Sigma-Aldrich, Oakville, Canada), pH 7.6 with 0.05% Tween-20 (TBST; Fisher Scientific, Markham, Canada). Primary monoclonal antibody against PRRSV nucleocapsid N protein (SDOW17, Rural Technologies Inc., Brookings, USA) was diluted 1:200 in antibody diluent (Dako) and placed on tissue sections overnight at 4 °C in a humidified chamber. Envision + System-HRP (Dako) anti-mouse secondary antibody containing 2% normal swine serum (Life Technologies) was applied to sections for 45 min at RT. The signal was revealed using 3-amino-9-ethylcarbazole (AEC) chromogen (Dako) for 15 min and sections were counterstained with Mayer’s hematoxylin (Fisher Scientific). Coverslips were applied to slides using Glycergel (Dako) mounting medium. Negative controls consisted of uterine tissues obtained from non-infected gilts.

### CD163, CD169 and cathepsin immunohistochemistry

IHC for CD163 and CD169 was performed as previously described [26, 27] using rabbit polyclonal antibodies directed against human CD163 at dilution 1:100 (ab87099, Abcam, Toronto, Canada) and mouse monoclonal antibodies directed against human CD169 (clone HSn 7D2) at dilution 1:50 (NB600-534, Novus Biologicals, Oakville, Canada). IHC for cathepsin was performed using mouse monoclonal antibodies directed against human cathepsin L + V at dilution 1:100 (ab6314, Abcam). Antigen retrieval was performed in 10 mM citrate buffer (pH 6.0) at 100 °C for 10 min. Using a Dako Automated Immunostainer and TBST for washing buffer, the antigen signals were amplified using Envision + System-HRP with 2% normal swine serum for 45 min and AEC chromogen for 10 min. Slides were counterstained with Mayer’s hematoxylin. Pig fetal lung and lymph node were used for the positive controls for CD163 and CD169 IHC. A normal pig uterus with placenta was used for the cathepsin positive control. Normal rabbit serum was used in place of primary antisera for the negative control.

### Image analysis

Quantitative analyses of immunohistochemical staining for PRRSV, CD163, CD169, and cathepsin positive areolae were performed using Image-Pro Plus version 7 software (Media Cybernetics, Inc., Rockville, USA). Ten microscopic fields of the endometrium, captured using a 20× microscope objective lens, each representing 1 mm^2^ area (10 mm^2^ total/slide) were randomly selected from each image of the uterine-placental tissue. Thereafter, multiple polygonal fields of fetal placenta comprising 3–4 mm^2^ in total were randomly selected from the same image at the same magnification. Inside these chosen fields, the total numbers of PRRSV, CD163 and CD169 immunopositive cells were manually counted. The total number of areolae was determined by manually counting the regions of cathepsin immunopositive staining across the entire maternal–fetal interface. All counts were expressed as a number per 1 mm^2^ area for statistical analyses.

### Statistical analysis

Separate statistical analyses were performed for each of the objectives of this study using Stata 13 (StataCorp LP, College Station, USA). To determine if numbers of CD163 and CD169 positive macrophages, and cathepsin positive areolae in the endometrium and fetal placenta differed among PRRS viral load groups (negative, low, high), separate two-level, linear mixed-effects regression models were developed. For these models, numbers of CD163 and CD169 positive macrophages in the endometrium and fetal placenta were zero-skewness log (lnskew0) transformed to ensure that model assumptions of linearity and homogeneity were not violated. Secondly, the potential relationships among numbers of CD163 and CD169 positive macrophages in the endometrium and the fetal placenta, numbers of cathepsin positive areolae at the MFI, and PRRS viral load in the fetal thymus were determined by using a two-level, zero-inflated Poisson regression model. For this model, PRRSV RNA concentration in fetal thymus (target copies per mg tissue) was converted into a count variable, with each successive count representing a one log_10_ increase in RNA concentration. Based on these results, the relationship between viral load in fetal thymus and the numbers of CD163^+^ and CD169^+^ macrophages in endometrium and fetal placenta was further explored using separate single level proportional odds models (a two-level model was not required) for which PRRSV RNA concentration in fetal thymus was categorized as negative (not detected), low (0 < log_10_ copies per mg <5) and high (log_10_ copies per mg ≥5), and new variables representing the ratio of ${\text{CD163}}_{\text{endo}}^{ + }$:${\text{CD163}}_{\text{plc}}^{ + }$ and ${\text{CD169}}_{\text{endo}}^{ + }$:${\text{CD169}}_{\text{plc}}^{ + }$ were created. For these proportional odds models, only fetuses from PRRSV infected gilts were included. All two-level models accounted for clustering by litter of origin by including gilt as a random effect. Linear mixed models were assessed for normality and homogeneity of residuals. The proportional odds model was assessed for non-violation of the proportional odds assumption using the Brant test. Count models were assessed by evaluating how well the models predicted raw data, and where possible, evaluating the degree of over-dispersion. *P* value was deemed significant at 0.05 a priori.

## Results

### Distribution of immunopositive cells within the maternal–fetal interface

**Figure 1:**
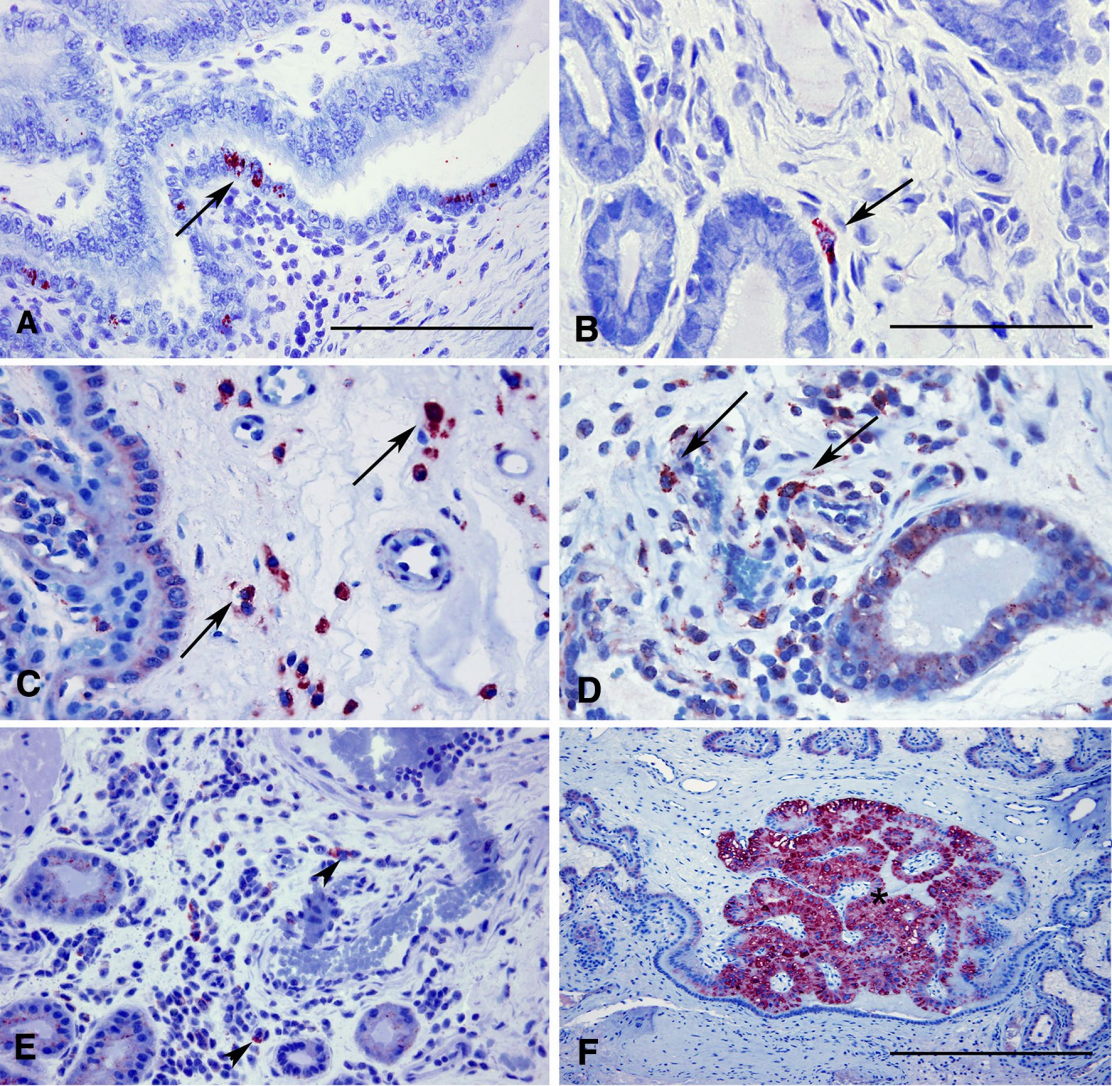
**IHC for PRRSV, CD163, CD169, and cathepsin in the PRRSV-infected uterine-fetal placental tissues.** **A** Strong immunopositive staining for PRRSV of uterine epithelial cells (arrow) of areola from PRRSV-infected pregnant gilt. IHC for SDOW17, bar = 200 μm **A** and **E**. **B** PRRSV positive immunostained macrophage-like cell (arrow) in the endometrium of PRRSV-infected pregnant gilt. IHC for SDOW17, bar = 100 μm **B**–**D**. **C** Strong cytoplasmic immunopositive staining for CD163 of macrophages (arrows) in the chorioallantois of PRRSV-infected pregnant gilt. IHC for CD163. **D** Increased numbers of CD163 macrophages in highly inflamed areas (arrows) in the *lamina propria* of endometrium from the uterus of PRRSV-infected pregnant gilt. IHC for CD163. **E** Positive CD169 cells (arrowheads) in the *lamina propria* of endometrium from the uterus of PRRSV-infected pregnant gilt. IHC for CD169. **F** Cathepsin immunopositive stained areola (asterisk) at the uterus-fetal placenta interface from PRRSV-infected pregnant gilt. IHC for Cathepsin, bar = 500 μm.

PRRSV immunopositive cells were detected in all sections of MFI obtained from PRRSV infected gilts. The majority of samples demonstrated strong PRRSV immunopositivity of cells located primarily at the endometrial placental junction (mean 8.6 ± 10.8 cells per mm^2^). PRRSV immunopositive cells at these histological sites were closely associated with inflammatory cell infiltrates and had cytological features suggestive of tissue macrophages. Occasionally PRRSV antigen was clearly present in the uterine superficial glandular epithelial cells of the areolae (Figure 1A). In the endometrium, PRRSV immunopositive macrophages were rare and located away from blood vessels in the *lamina propria* (mean 1.7 ± 2.2 cells per mm^2^) (Figure 1B). Additionally, a remarkable finding in the endometrium was strong, albeit occasional, PRRSV immunopositive staining of epithelial cells of uterine glands. Rare PRRSV-infected macrophages were found in the fetal placenta residing in proximity to the MFI of PRRSV-infected gilts. PRRSV-infected cells were not detected in the MFI of sham inoculated gilts.

IHC for CD163 and CD169 revealed the largest number of CD163 immunopositive macrophage-like cells (mean 212.3 ± 82.6 per mm^2^) resided in the fetal placenta in close proximity to maternal and fetal microvilli interdigitation (Figure 1C), while the number of CD169 immunopositive cells observed in the fetal placenta were significantly lower (mean 69.8 ± 71.6 per mm^2^). In the endometrium, markedly lower numbers of CD163 immunopositive cells (mean 34.2 ± 27.2 per mm^2^) and CD169 immunopositive cells (mean 15.6 ± 15.7 per mm^2^) were observed multifocally in the *lamina propria*, occasionally in the blood vessel walls, and always interspersed throughout inflammatory cell infiltrate (Figures 1D and E). IHC of cathepsin revealed strong positive cytoplasmic staining of collections of trophoblastic cells forming distinct placental structures at the maternal–fetal interface most consistent with areolae (Figure 1F). Regardless of the infection status of pregnant gilts, numbers of areolae counted at the MFI of each uterine tissue section were relatively stable (mean 3.5 ± 1.9).

### Relationship of cell counts to viral load

**Figure 2:**
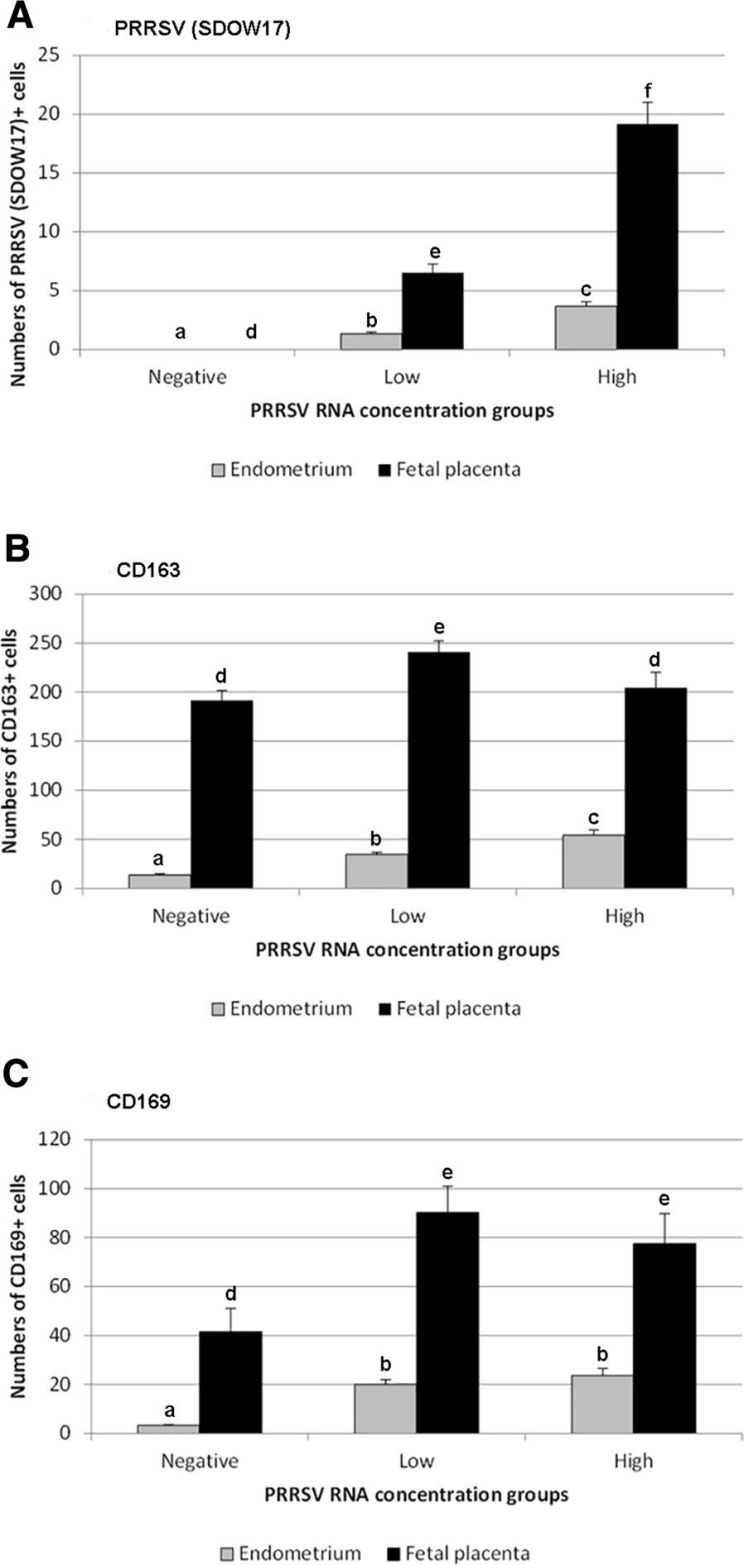
**Mean numbers of PRRSV, CD163 and CD169 positive cells per 1** **mm** ^**2**^ **of endometrium and placenta.** **A** Mean numbers of PRRSV (SDOW17) positive cells per 1 mm^2^ of the endometrium and fetal placenta. **B** Mean numbers of CD163 positive cells per 1 mm^2^ of the endometrium and fetal placenta. **C** Mean numbers of CD169 positive cells per 1 mm^2^ of the endometrium and fetal placenta. Superscript letters (a, b, c or d, e, f) indicate significant differences (*P* < 0.05) between PRRSV viral load groups. Error bars represent standard error.

Statistical analyses of the results of IHC experiments revealed significantly higher numbers of immunopositive cells in the endometrium and fetal placenta obtained from PRRSV infected gilts than negative control gilts for all markers (Figures 2A–C). Only exception from these results was lack of difference in the numbers of CD163^+^ cells in the fetal placenta between negative control gilts and infected high viral load group (Figure 2B). Statistical differences were also found when low and high viral load groups were compared in terms of the numbers of PRRSV-infected cells present in the endometrium and the fetal placenta at the MFI (*P* < 0.05) (Figure 2A). However, high PRRSV load group was characterized by significantly higher numbers of CD163^+^ cells in the endometrium, but lower numbers in fetal placenta, compared to the low PRRSV load group (Figure 2B). Even though no significant differences were found in the numbers of CD169^+^ cells in the endometrium and fetal placenta between low and high PRRSV load groups, a similar trend of increased of cells in the endometrium and decreased cells in fetal placenta as seen with CD163^+^ cells was observed (Figure 2C). Numbers of cathepsin-L immunostained areolae across the MFI were also not statistically different between PRRS viral load groups (data not shown).

**Figure 3:**
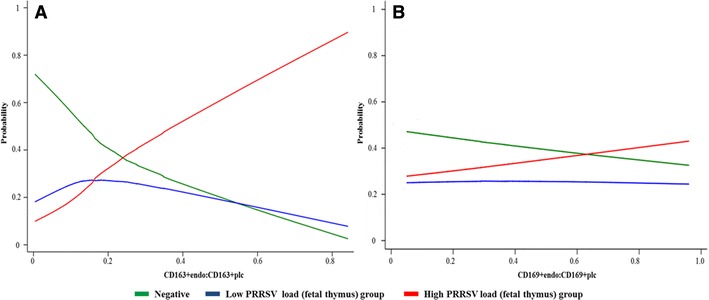
**Relationship between PRRSV RNA concentration in fetal thymus and the ratio of** ${\mathbf{CD163}}_{{{\mathbf{endo}}}}^{{\mathbf{ + }}}$ **:** ${\mathbf{CD163}}_{{{\mathbf{plc}}}}^{{\mathbf{ + }}}$ **and** ${\mathbf{CD169}}_{{{\mathbf{endo}}}}^{{\mathbf{ + }}}$ **:** ${\mathbf{CD169}}_{{{\mathbf{plc}}}}^{{\mathbf{ + }}}$ **.** Results of single level proportional odds models. The probability of a fetus being in each of three PRRSV RNA concentration categories is shown: negative (green), low (blue; 0 < log_10_ copies per mg <5) and high (red; >5 log_10_ copies per mg). **A** Based on ratio of ${\text{CD163}}_{{{\text{endo}}}}^{{{ + }}}$:${\text{CD163}}_{{{\text{plc}}}}^{{{ + }}}$ cells (*P* = 0.001), the probability of a fetuses being in the high viral load category (red line) increases dramatically as ${\text{CD163}}_{{{\text{endo}}}}^{{{ + }}}$:${\text{CD163}}_{{{\text{plc}}}}^{{{ + }}}$ ratio increases (opposite true for negative and low viral load categories). **B** Based on ratio of ${\text{CD169}}_{{{\text{endo}}}}^{{{ + }}}$:${\text{CD169}}_{{{\text{plc}}}}^{{{ + }}}$ cells (*P* = 0.07) a similar trend is noted but the strength of association is weak and may not be biologically relevant.

**Figure 4:**
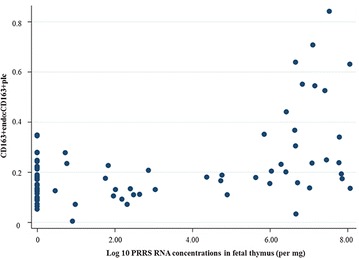
**Relationship between ratio of** ${\mathbf{CD163}}_{{{\mathbf{endo}}}}^{{\mathbf{ + }}}$ **:** ${\mathbf{CD163}}_{{{\mathbf{plc}}}}^{{\mathbf{ + }}}$ **and PRRSV RNA concentration in fetal thymus.** Scatter plot of ratio of numbers of CD163^+^ cells in the endometrium and CD163^+^cells in fetal placenta (Y axis)_,_ and PRRSV RNA concentration (log_10_ copies/mg) in fetal thymus (X-axis). Each dot represents one fetus. Results indicate that PRRSV RNA concentration in fetal thymus is positively associated with ratio of ${\text{CD163}}_{{{\text{endo}}}}^{{{ + }}}$:${\text{CD163}}_{{{\text{plc}}}}^{{{ + }}}$ (all fetuses with viral load less than 6 log_10_ per mg had endo:plc ratios less than 0.4).

The numbers of CD163^+^ and CD169^+^ macrophages in the endometrium and fetal placenta and cathepsin positive areolae were assessed as potential predictors of PRRSV viral load in fetal thymus. After a backwards-stepwise elimination, only numbers of CD163^+^ macrophages in endometrium and fetal placenta were related to PRRS viral load in the fetal thymus (*P* < 0.001). More specifically, increased PRRSV RNA concentration (log_10_ copies/mg) in the fetal thymus was associated with increased numbers of CD163^+^ macrophages in the endometrium and decreased numbers of CD163 macrophages in placenta (Table 1). The dichotomous relationships between fetal viral load and CD163 cells in placenta and endometrium, along with the trend of decreased CD163^+^ and CD169^+^ cell numbers in fetal placenta between low and high viral groups, prompted further investigation. To this end, the ratio of ${\text{CD163}}_{\text{endo}}^{ + }$:${\text{CD163}}_{\text{plc}}^{ + }$ and ${\text{CD169}}_{\text{endo}}^{ + }$:${\text{CD169}}_{\text{plc}}^{ + }$ cells were assessed as predictors of viral load in fetal thymus (categorized as negative, low of high). In separate proportional odds models, viral load in the fetal thymus was found to be very strongly related to ${\text{CD163}}_{\text{endo}}^{ + }$:${\text{CD163}}_{\text{plc}}^{ + }$ ratio (coef. 7.1 ± 2.1, 95% CI 3.1, 11.2; *P* = 0.001) (Figure 3A). In fact, all fetuses with thymic PRRSV RNA concentration less than 6 log_10_ copies per mg had ratios less than 0.4 (Figure 4). PRRSV load in the fetal thymus was weakly related to ${\text{CD169}}_{\text{endo}}^{ + }$:${\text{CD169}}_{\text{plc}}^{ + }$ ratio (coef. 0.73 ± .4, 95% CI −.06, 1.5; *P* = 0.07) revealing that high viral load may be associated with higher ratio (increased numbers of CD169^+^ cells in the endometrium and decreased numbers in fetal placenta), but with clearly significantly lower influence and confidence compared to CD163^+^ cells (Figure 3B).

**Table 1:** **Association of CD163 positive cells in the endometrium and placenta and PRRSV RNA concentration in fetal thymus**

PRRS RNA concentration in fetal thymus count^e^	Coefficient (SE)	95% CI	*P* values
Continuous model (relationship of cell numbers to PRRSV concentration in fetal thymus)			
Numbers of CD163 positive cells per 1 mm^2^ of endometrium	0.004 (0.002)^a^	0.0009, 0.007	0.011
Numbers of CD163 positive cells per 1 mm^2^ of fetal placenta	−0.002 (0.001)^b^	−0.003, −0.0006	0.005
Inflated (logit) model^f^ (relationship of cell numbers to likelihood of PRRSV concentration in fetal thymus being equal to zero (PRSV negative)			
Numbers of CD163 positive cells per 1 mm^2^ of endometrium	−0.044 (0.012)^c^	−0.067, −0.021	<0.001
Numbers of CD163 positive cells per 1 mm^2^ of fetal placenta	0.006 (0.003)^d^	0.0004, 0.011	0.033

Results of zero-inflated Poisson regression model.

^a^For each one unit increase in the numbers of CD163 positive cells/mm^2^ of endometrium, PRRSV RNA concentration in fetal thymus increases by 0.004 log_10_ copies/mg.

^b^For each one unit increase in the numbers of CD163 positive cells/mm^2^ of fetal placenta PRRSV RNA concentration in the fetal thymus decreases by 0.002 log_10_ copies/mg.

^c^For each one unit change in the numbers of CD163 positive cells/mm^2^ of endometrium the odds of PRRSV RNA concentration in fetal thymus being equal to 0 (zero) decreases 1.045 times (e^−0.044^).

^d^For each one unit change in the numbers of CD163 positive cells/mm^2^ of fetal placenta the odds of PRRSV RNA concentration in the fetal thymus being equal to 0 (zero) increases 1.006 times (e^0.006^).

^e^Based on the distribution of data, a count model was used after categorizing PRRSV RNA concentration into nine 1 − log_10_ replicates, from 0 to 9 log_10_ copies/mg.

^f^The logit portion of the model predicts the odds of a fetus being negative (PRRSV RNA concentration = 0) from the population of fetuses from infected and negative control gilts.

## Discussion

The goal of this study was to evaluate the associations between the numbers of CD163 and CD169 positive cells in the endometrium and fetal placenta, cathepsin positive areolae at the MFI, and PRRSV viral load in the MFI and fetal thymus. These results provide improved insights into the events occurring at the maternal–fetal interface during type 2 PRRSV infection and help clarify the pathogenesis of PRRSV transplacental infection and induced reproductive failure. Previous studies [28, 29] as well as our observations in a related histopathology study of type 2 PRRSV infection in pregnant gilts [24] confirmed that type 2 PRRSV infection causes significant microscopic lesions in the uterus and fetal placenta at the maternal–fetal interface. Consequently, we hypothesized that in addition to CD169 and CD163^+^ cells, PRRSV in utero infection is influenced by other factors involving epithelial cells of MFI and areolae.

The findings of our first objective confirmed that there was a significant increase in the numbers of CD163^+^ and CD169^+^ macrophages in the endometrium and the fetal placenta in PRRSV-infected versus non-infected pregnant gilts. This finding was not unexpected because large numbers of these cells represent a significant portion of the cellular infiltrate in the highly inflamed areas of *lamina propria* suggesting a role in the innate immune response to PRRSV infection. On the other hand, differences in these cell numbers were much less consistent when the low and high PRRSV groups were compared. For example, only the numbers of CD163^+^ cells in endometrium were significantly increased in high versus low viral load groups. Nevertheless, this inconsistent relationship between cell counts and viral load reported herein is in the agreement with the results of our previously reported PRRS histopathologic evaluation [24] in which it was demonstrated that the severity of endometrial inflammation was not associated with PRRSV viral load in the uterus at 21 dpi.

Not only did cell counts in fetal placenta differ between negative and high PRRS viral load groups, they markedly diverged from the results obtained from the endometrium. In particular, this applied to the numbers of CD163^+^ cells in placenta, which did not significantly differ in non-infected gilts compared to gilts with high PRRS viral load at the MFI. A possible explanation for this finding could be the presence of a residential population of fetal tissue macrophages, which in human placenta are known as Hofbauer cells. This population of histiocytes found in human villous mesenchyme and amniochorion on the fetal side of the uteroplacental unit is relatively constant, and can constitute nearly all macrophages in this region [30]. It is believed these macrophages are involved in the prevention of the transmission of the pathogens from the mother to the fetus (vertical transmission) and early placental development [31]. Importantly, Hofbauer cells strongly express CD163, CD68, and CD206 [31–33].

The presence of Hofbauer cells in the porcine fetal chorioallantois has not been reported to date, but in this present study, we observed large numbers of histiocytes with cytological features resembling Hofbauer cells and expressing strong cytoplasmic CD163 immunopositivity residing along the chorionic villi of the fetal placenta in both infected and non-infected gilts. Moreover, the number of CD163^+^ cells in placenta was negatively associated with viral load in fetal thymus, and increased numbers were significantly related to increased odds of a fetus being virus negative. This finding implies that CD163^+^ tissue macrophages in the placenta may have a potentially significant role in type 2 PRRSV infection of fetus. While numbers of CD163^+^ tissue macrophages in the endometrium are concomitant with uterine viral load, the numbers of CD163^+^ cells in the fetal placenta might decrease subsequent to transplacental PRRSV infection.

To explore this potential dichotomy further, we assessed the relationship between viral load in fetal thymus and the ratio of CD163^+^ cells present in endometrium and fetal placental (CD163_endo_:CD163_plc_) based on our hypothesis that a high ratio (reflecting increased macrophage numbers and infection pressure in uterus and low placental immune surveillance) would be associated with high fetal viral load. As anticipated, viral load in fetal thymus increased very substantially as the CD163^+^ endo:plc ratio increased. In addition, the probability of fetal thymus being PRRSV virus negative decreased dramatically in a nearly linear manner as CD163_endo_:CD163_plc_ increased (Figure 3A). Thus, large numbers of CD163^+^ macrophages in placenta, particularly in the presence of low CD163^+^ macrophages in the endometrium, may represent an exciting, yet unexplored mechanism of PRRSV resistance in late gestation fetuses.

However, in order to draw this conclusion, at least one criterion would have to be met; that is the porcine analogs of Hofbauer cells at the fetal placenta are not susceptible to PRRSV infection and subsequent viral replication. Unfortunately, current knowledge on the properties of this porcine cell population regarding the PRRSV susceptibility is poor; therefore, future studies in this area are highly warranted. On the other hand, fetal placenta (chorioallantois) is confirmed to be highly susceptible to type 1 PRRSV infection demonstrating markedly larger numbers of PRRSV-positive cells than the endometrium at 10 dpi [18]. Although type 1 and type 2 PRRS viruses are genetically distinct with differences in pathogenicity in vitro and in vivo [34], it can also be hypothesized that by collecting the fetal placenta 21 dpi in our study we detected a decrease in the numbers of CD163^+^ cells as a result of the cytopathic effect of PRRSV on a highly susceptible population of cells. Potential evidence for this hypothesis is similar trend of decrease in CD163^+^ and CD169^+^ cells in the fetal placenta between low and high viral load group (Figures 2B, C). Although, our previous analyses conducted for the same animal experiment confirmed a significant positive association between the PRRSV RNA concentration in fetal thymus and MFI [25, 35], along with similar proportion of the litter and MFI samples that tested PRRSV qRT-PCR positive [24], we can not completely exclude the possibility that differences in PRRSV load in the fetuses and fetal placenta in the MFI partially reflect different time points of infection. Therefore, higher PRRSV RNA concentration in the fetal tissue could result in more pronounced cytopathic effect on susceptible cells. In this view, our findings could also suggest that instead of a protective role, placental CD163^+^ cells may play a role in transplacental infection or viral replication in the fetal compartment following infection. In other words, once PRRSV transmits transplacentally, the resident CD163^+^ cells become “fertile soil” for viral propagation and spread to the fetal organs. Therefore, it is essential to conduct future studies aimed at determining the susceptibility of porcine placental macrophages to PRRSV infection. Increase in the numbers of CD163^+^ and also CD169^+^ cells in the endometrium could be explained by an influx of blood monocytes differentiating into both cell populations as a result of a marked inflammatory process driven not only by PRRSV, but also by significant tissue damage associated with vasculitis, necrosis of uterine glands, and apoptosis. By contrast, the limited capacity of developing fetal hematopoietic tissue to replenish following challenge [36] could contribute to the decrease of PRRSV susceptible macrophages in the chorioallantois.

Unlike CD163^+^ cells, numbers of CD169^+^ cells were significantly different between infected and uninfected groups, but no significant difference was found between high and low viral groups. Both cell types demonstrated similar trend of increase in the endometrium and decrease in placenta during the PRRSV infection in the MFI. However, we found that CD169^+^ cells in endometrium and fetal placenta were not related to PRRSV load in fetal thymus (Table 1). Moreover, the ratio of CD169^+^ cells in endometrium and fetal placenta was only weakly associated with viral load in fetal thymus (Figure 3B). Although a few previously published reports indicate the importance of CD169 as a receptor mediating cell entry for type 1 PRRSV [8, 15], a recent study in the transgenic pigs [16] confirmed that intact CD169 receptor is not required for productive type 2 PRRSV infection. The importance of CD163 rather than CD169 for type 2 PRRSV viral replication was corroborated in pigs with an edited (non-functional) CD163 where viral replication did not occur [17]. Our results tend to agree with the latter studies suggesting CD163^+^ macrophages play a potentially more important role in PRRSV infection of the MFI than do CD169^+^ macrophages. That being said, more research is clearly needed to determine the exact role of CD163^+^ placental macrophages, if they are also relevant for other reproductive pathogens in pigs and other animals (including humans), and the potential effects of CD163^−/−^ gene editing in a pregnant animal.

Because in the present study PRRSV antigen has been occasionally detected in the uterine epithelial cells of the areolae, and considering the active role the areolae have in nourishment of the fetus, we also evaluated the potential association between numbers of cathepsin positive placental areolae and PRRSV load in the fetus. While we were unable to evaluate the size (or area) of the areolae in the present study, the numbers of areolae were not significantly associated with PRRSV load in the fetal thymus.

PRRSV IHC studies on the uterine tissue with fully attached fetal chorioallantois have been rarely reported in the past. In the present study, the presence of the PRRS virus antigen in uterine tissues and fetal placentae was infrequent and rarely localized in the inflammatory cells of the *lamina propria*. This unexpected finding could be due to the time point used for the collection of samples which at 21 dpi was past peak. Previous experiments also confirmed lesser numbers of type 1 PRRSV immunopositive cells in the endometrium of the sows euthanized at 20 dpi than in those euthanized at 10 dpi [18, 23]. Therefore, using IHC for detection of PRRSV antigen in the MFI is optimal in the early time points of infection, but in our study other experimental activities necessitated collection at 21 dpi. On the other hand, the PRRSV replication in the fetal lymphoid organs can continue and persist after birth [37].

The largest numbers of cells staining with PRRSV antigen in the cytoplasm were found at the MFI resembling histiocytes, and to a lesser degree but surprising, in uterine epithelial cells and the rare fetal trophoblastic cells. PRRSV immunopositivity of uterine and trophoblastic epithelial cells along with occasional moderate PRRSV immunostaining of the glandular epithelium of the uterine glands were novel findings in this study. PRRSV infection of nasal, bronchiolar and alveolar epithelium has been reported before, but the mechanism of the PRRSV infection of these cell types remains unexplained [2, 6, 38]. Nevertheless, it has been confirmed that some epithelial cells such as St-Jude porcine lung cells are susceptible to in vitro PRRSV infection due to the expression of receptor CD151 [39]. CD151 receptor has been also implicated in PRRSV infection of porcine endometrial endothelial cells, where it is believed to act as alternative receptor along with CD169 [40]. Additionally, syndecan-4, which is heparan sulfate proteoglycan, is confirmed to be required in the PRRSV attachment to MARC-145 cells [41]. Heparan sulfate proteoglycans are present on the epithelial and endothelial cells and are confirmed to bind to M and N proteins of the PRRSV [42]. Another important finding from our PRRSV immunohistochemical analysis was the detection of PRRSV antigen in the smaller numbers of macrophage-looking cells present in the fetal chorioallantois in the proximity of MFI suggesting potential cell-associated virus spread from the endometrium to the fetal membranes.

In summary, the results of this study confirmed significant increases in the numbers of PRRSV^+^, CD163^+^ and CD169^+^ cells at the MFI during late gestation, type 2 PRRSV infection in pregnant gilts. The relationships between numbers of CD163^+^ and CD169^+^ cells in the endometrium and fetal placenta, and PRRSV viral load in the fetal thymus suggests the more important role of CD163 expressing cells, which provides additional evidence of their potential role in type 2 PRRSV fetal infection.

## Abbreviations

AEC: 3-amino-9-ethylcarbazole
AREB: animal research ethics board
CD: cluster of differentiation
DNA: deoxyribonucleic acid
IHC: immunohistochemistry
MFI: maternal–fetal interface
PAM: pulmonary alveolar macrophages
PCR: polymerase chain reaction
PIM: pulmonary intravascular macrophages
PRRS: porcine reproductive and respiratory syndrome
PRRSV: porcine reproductive and respiratory syndrome virus
RT-qPCR: quantitative real-time PCR assay
RNA: ribonucleic acid
TCID_50_: 50% tissue culture infective dose
